# Elevated Risk of Complications in Patients Receiving Dual Antithrombotic Therapy Undergoing Hepatectomy: A Single‐Center Audit of 749 Cases

**DOI:** 10.1002/ags3.70160

**Published:** 2025-12-29

**Authors:** Haruki Mori, Hiromitsu Maehira, Nobuhito Nitta, Takeru Maekawa, Toru Miyake, Sachiko Kaida, Masatsugu Kojima, Katsushi Takebayashi, Takeshi Sonoda, Masaji Tani

**Affiliations:** ^1^ Department of Surgery Shiga University of Medical Science Otsu Shiga Japan

**Keywords:** antithrombotic therapy, dual therapy, hemorrhagic complications, hepatectomy, thromboembolic events

## Abstract

**Aim:**

Increase in surgical patients undergoing antithrombotic therapy (ATT) has intensified the concerns regarding perioperative bleeding and thromboembolic complications. This study evaluated the safety of hepatectomy in patients receiving ATT, with a focus on those receiving dual therapy.

**Methods:**

We retrospectively analyzed 749 patients who underwent elective hepatectomies between 2011 and 2024. Among them, 140 received ATT according to the Japanese guidelines, and 98 were matched 1:1 with non‐ATT controls using propensity scores. The primary outcome was major morbidity, defined as Clavien–Dindo ≥grade III; intraoperative blood loss and transfusion requirement were secondary outcomes. Outcomes were also compared across the ATT regimens, including dual therapy (antiplatelet plus anticoagulants).

**Results:**

Compared with the non‐ATT group, the ATT group had a significantly longer operative time (median, 318 vs. 250 min; *p* = 0.019), while blood loss (355 vs. 300 mL; *p* = 0.460), transfusion rate (19.4% vs. 23.5%; *p* = 0.602), and severe complication rate (32.7% vs. 22.4%; *p* = 0.150) were not significantly different. Post‐hepatectomy liver failure (PHLF) incidence (16.3% vs. 13.3%) and median hospital stay (13 vs. 12 days) were also comparable. In subgroup analysis, patients receiving dual therapy exhibited higher complication rates, including hemorrhagic events (30%), Clavien–Dindo≥grade III (40%), and PHLF (50%), exceeding the rates seen in monotherapy groups.

**Conclusion:**

Hepatectomies can be safely performed in patients receiving well‐managed ATT. However, dual therapy was associated with higher rates of perioperative complications, including bleeding and PHLF, compared with monotherapy or no ATT, underscoring the need for heightened caution and individualized management in this high‐risk group.

## Graphical abstract

This single‐center retrospective study evaluated perioperative outcomes after hepatectomy in 749 patients, including 140 receiving ATT, using propensity score matching to compare ATT and non‐ATT cohorts. Under standardized perioperative management, ATT did not increase major bleeding, transfusion, or severe morbidity overall; however, within the ATT subgroup, patients on dual therapy (combined antiplatelet and anticoagulant agents) exhibited markedly higher rates of hemorrhagic complications, post‐hepatectomy liver failure, and Clavien–Dindo grade III or higher complications than those on monotherapy. These findings suggest that hepatectomy is generally safe in patients receiving well‐managed ATT, whereas dual therapy identifies a particularly high‐risk group requiring meticulous perioperative management.

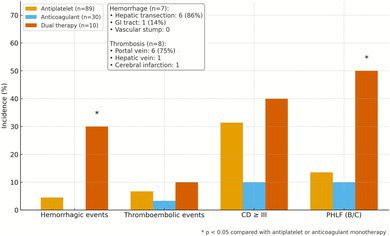

## 1 Introduction

Antithrombotic therapy (ATT), including antiplatelet and anticoagulant agents, is widely prescribed to prevent thromboembolic events in patients with cardiovascular and cerebrovascular diseases. With an aging population, the number of surgical candidates receiving ATT is increasing worldwide. While the perioperative management of ATT in endoscopic or low‐risk surgical procedures has been addressed in multiple guidelines, such as those from the Japanese Circulation Society [1] and the American College of Chest Physicians [2], major abdominal surgeries, including hepatectomies, remain particularly challenging due to the high risk of bleeding and liver failure [3, 4].

Hepatectomy, especially anatomical resection, involves a complex vascular anatomy and substantial parenchymal transection, resulting in a non‐negligible risk of perioperative complications. For patients receiving ATT, surgical teams must balance the risks of bleeding from continued therapy and thromboembolism from withdrawal. Although some reports suggest that elective gastrointestinal and urological surgeries can be safely performed under ATT [5], evidence for hepatectomy remains sparse and inconclusive.

Furthermore, there is increasing awareness of ethnic variability in thromboembolic tendencies. Asian patients, including Japanese populations, generally demonstrate a lower baseline risk of venous thromboembolism compared with Western populations [6, 7]. This raises concerns about the applicability of Western guideline‐based strategies to Eastern surgical cohorts.

Therefore, we conducted a propensity score‐matched analysis of a large single‐center cohort to evaluate the perioperative risks associated with ATT in patients undergoing hepatectomy. This study uniquely categorized ATT regimens based on real‐world clinical practice, including antiplatelet, anticoagulant, and dual therapies, and examined detailed complication profiles, such as the site of bleeding, location of thromboembolic events, and incidence of post‐hepatectomy liver failure (PHLF), to better inform risk stratification and perioperative management.

## 2 Methods

### 2.1 Study Design

We retrospectively reviewed the electronic medical records of patients who underwent hepatectomy between January 2011 and September 2024 at Shiga University of Medical Science Hospital. Patients who underwent hepatectomy with concomitant resection of other organs (*n* = 36) and those with unclear information regarding the use of antithrombotic agent use (*n* = 25) were excluded. After exclusion, 749 patients were identified, and their clinical data were reviewed. All patients provided written informed consent prior to treatment. This study was approved by the Human Ethics Review Committee of Shiga University of Medical Science (approval number: 2017–170) and conducted in accordance with the principles of the Declaration of Helsinki. Informed consent was obtained from all patients or their family members during outpatient visits.

Patients were categorized into two groups according to their regular use of ATT. To assess the impact of hepatectomy in patients undergoing ATT, surgical outcomes and the incidence of postoperative complications, including hemorrhagic and thromboembolic complications, were compared between the two groups.

### 2.2 Surgical Procedure

After review by a multidisciplinary board, all liver disease cases were assessed by liver surgeons to determine resectability and the most appropriate surgical procedure. Preoperative cholangiography was not usually performed. Intraoperative ultrasonography was performed to determine the location of the lesion and line of parenchymal resection. Liver parenchymal transection was performed using an ultrasonic dissector and Cavitron Ultrasonic Surgical Aspirator. Anatomical resection was defined as hepatectomy involving subsegmentectomy or larger, whereas non‐anatomical resection included partial resection.

### 2.3 Data Collection and Definitions

Data on demographic characteristics, preoperative laboratory values, intraoperative variables, and postoperative outcomes were systematically collected. Postoperative complications were classified according to the Clavien–Dindo grading system. PHLF was defined according to the criteria established by the International Study Group of Liver Surgery (ISGLS).

### 2.4 Perioperative Management of Antithrombotic Drugs

Perioperative management of patients receiving ATT was conducted in accordance with the 2020 Guideline Focused Update on ATT in Patients With Coronary Artery Disease issued by the Japanese Circulation Society (JCS) [1]. Decisions regarding continuation, temporary discontinuation, or bridging of antithrombotic agents, including antiplatelet drugs and anticoagulants, were made through a multidisciplinary consensus involving surgeons, cardiologists, and anesthesiologists. The detailed drug‐specific perioperative management protocol is provided in Supplementary Methods.

### 2.5 Propensity Score Matching and Statistical Analysis

To reduce potential selection bias between the ATT and non‐ATT groups, propensity score matching (PSM) was performed using a 1:1 nearest‐neighbor matching algorithm without replacement, with the caliper width of 0.01. Matching was conducted based on clinically relevant covariates including age, sex, primary disease, repeat liver resection, surgical approach (open or laparoscopic), liver resection procedure (anatomical or partial), liver function parameters (albumin, bilirubin) and biliary reconstruction. Specific indications for ATT, such as coronary artery disease, atrial fibrillation, or prior ischemic stroke, were not included as covariates in the propensity score model to avoid overfitting and model instability, given the relatively small number of ATT patients, particularly those receiving dual therapy. The propensity score model was therefore limited to core demographic and surgical variables, although residual confounding by indication may persist. The adequacy of covariate balance between matched groups was assessed using standardized mean differences (SMDs), with an SMD < 0.1 indicating a negligible imbalance.

Categorical variables were expressed as numbers and percentages, while continuous variables were reported as medians with interquartile ranges. For comparisons between the ATT and non‐ATT groups, intergroup differences were evaluated using the chi‐square test or Fisher's exact test for categorical variables and the Mann–Whitney U test for continuous variables. Within the ATT group, perioperative binary outcomes (e.g., hemorrhagic complications, thromboembolic events, PHLF, Clavien–Dindo ≥ grade III complications, and transfusion requirement) were compared among the three regimens (antiplatelet monotherapy, anticoagulant monotherapy, and dual therapy) using chi‐square tests for global comparisons. Continuous variables (intraoperative blood loss, operative time, and length of postoperative hospital stay) were compared among the three regimens using the Kruskal–Wallis test. When a global test was statistically significant (*p* < 0.05), post hoc pairwise comparisons focusing on dual therapy versus antiplatelet monotherapy and dual therapy versus anticoagulant monotherapy were performed using Fisher's exact test for categorical variables and the Wilcoxon rank‐sum test for continuous variables, and the resulting *p* values were adjusted for multiple testing with Bonferroni's method. All statistical analyses were performed using EZR (Easy R) version 1.55 (Saitama Medical Center, Jichi Medical University, Saitama, Japan), which is a graphical user interface for R (The R Foundation for Statistical Computing, Vienna, Austria). Statistical significance was defined as a two‐sided *p*‐value of < 0.05.

## 3 Results

### 3.1 Patient Characteristics Before and After Propensity Score Matching

In the original cohort, patients who received ATT were significantly older (76 vs. 69 years; *p* < 0.001), predominantly male (90.0% vs. 69.0%; *p* < 0.001), and more likely to have primary liver malignancy (75.0% vs. 49.5%; *p* < 0.001). They also exhibited lower serum albumin (3.8 vs. 4.0 g/dL; *p* < 0.001) and prothrombin activity (89% vs. 97%, *p* < 0.001), indicating compromised liver function.

After PSM, the 98 patients in each group were balanced for the covariates included in the propensity score model, namely age, sex, BMI, surgical method (open vs. laparoscopic: 45.9% vs. 43.9%; *p* = 0.886), procedure type (anatomical resection: 52.0% vs. 50.0%), liver function parameters (albumin, bilirubin), and disease distribution (primary vs. metastatic). The SMDs for all covariates were < 0.2 and most were < 0.1, which confirmed successful matching (Table 1).

**TABLE 1:** Patient characteristics before and after propensity score matching.

Variables	Non‐antithrombotic therapy (*n* = 609)	Antithrombotic therapy (*n* = 140)	*p*‐value	SMD	Non‐antithrombotic therapy (*n* = 98)	Antithrombotic therapy (*n* = 98)	*p*‐value	SMD
Age (years)	69 [62, 75]	76 [70.5, 80]	**< 0.001**	0.897	76 [70, 80]	76 [70, 80]	0.827	0.031
Sex			**< 0.001**	0.547			0.805	0.071
Male	420 (69.0)	126 (90.0)			90 (91.8)	88 (89.8)		
Female	189 (31.0)	14 (10.0)			8 (8.2)	10 (10.2)		
Body mass index (kg/m^2^)	22 [21, 25]	23.5 [21, 25]	0.100	0.169	23.7 [22.0, 26.0]	23.6 [21.5, 25.8]	0.100	0.093
Primary disease			**< 0.001**	0.519			0.265	0.160
Primary liver cancer/neoplasm	302 (49.5)	105 (75.0)			84 (85.7)	80 (81.6)		
Liver metastasis	213 (35.0)	24 (17.1)			13 (13.3)	15 (15.3)		
Others	94 (15.5)	11 (7.9)			1 (1.0)	3 (3.1)		
Preoperative laboratory data								
White blood cell count (μL)	5100 [4175, 6300]	5500 [4600, 6800]	0.055	0.193	5200 [3900, 6300]	5400 [4600, 6725]	0.171	0.215
Hemoglobin (g/dL)	12.9 [11.7, 14.1]	12.6 [11.1, 14.3]	0.155	0.184	13.2 [11.6, 14.4]	12.9 [11.2, 17.2]	0.306	0.161
Platelet count (x10^3^μL)	178 [135, 222]	169 [130, 211]	0.530	0.061	155 [110, 205]	166 [131, 205]	0.396	0.121
Albumin (g/dL)	4.0 [3.6, 4.2]	3.8 [3.5, 4.0]	**< 0.001**	0.419	3.8 [3.5, 4.1]	3.8 [3.5, 4.0]	0.787	0.039
Total bilirubin (mg/dL)	0.7 [0.5, 1.0]	0.6 [0.5, 0.9]	0.815	0.008	0.7 [0.5, 1.0]	0.7 [0.5, 0.9]	0.506	0.095
Aspartate aminotransferase (IU/L)	27 [21, 39]	28 [21, 44]	0.583	0.062	32 [24, 46]	28 [22, 46]	0.637	0.068
Alanine aminotransferase (IU/L)	22 [16, 36]	26 [16, 39]	0.678	0.034	28 [18, 44]	26 [16, 41]	0.566	0.082
Creatinine (mg/dL)	0.80 [0.67, 0.92]	0.87 [0.78, 1.09]	0.390	0.401	0.84 [0.77, 1.02]	0.86 [0.78, 1.08]	0.340	0.152
Prothronbin activity (%)	97 [88, 106]	89 [78, 100]	< 0.001	0.642	95 [86, 10.3]	92 [78, 102]	**0.014**	0.354
C‐reactive protein (mg/dL)	0.12 [0.10, 0.30]	0.15 [0.10, 0.40]	0.860	0.030	0.14 [0.07, 0.40]	0.13 [0.07, 0.35]	0.837	0.030
ICGR15 (%)	10.7 [6.5, 17.3]	11.7 [8.5, 19.6]	0.679	0.069	13.5 [8.9, 20.3]	11.6 [8.6, 18.9]	0.181	0.192
Repeat liver resection	141 (25.8)	34 (26.8)	0.789	0.001	31 (31.6)	26 (26.5)	0.529	0.113
Liver resection procedure			0.156	0.154			0.886	0.041
Anatomical resection	251 (41.2)	67 (47.9)			51 (52.0)	49 (50.0)		
Partial resection	358 (58.8)	73 (52.1)			47 (48.0)	49 (50.0)		
Surgical approach			0.403	0.075			0.886	0.041
Open laparotomy	324 (53.2)	69 (49.3)			45 (45.9)	43 (43.9)		
Laparoscopy	285 (46.8)	71 (50.7)			53 (54.1)	55 (56.1)		
Biliary reconstruction	36 (5.9)	10 (7.1)	0.585	0.084	4 (4.1)	6 (6.1)	0.747	0.093

*Note:* Left columns: before matching; right columns: after 1:1 matching between ATT and non‐ATT groups. Data are expressed as the median [IQR] and number (percent), except for age and body mass index, which are expressed as mean values ± standard deviation. Bold values denote statistical significance at the *p* < 0.05 level.

Abbreviation: ICGR15, indocyanine green retention rate at 15 min.

### 3.2 Details of Antithrombotic Regimens

Among the ATT prescriptions, antiplatelet agents are more commonly used than anticoagulants. Aspirin was the most frequently prescribed agent (44.4%), followed by clopidogrel (15.0%) and edoxaban (10.6%). The other drugs included warfarin (6.9%), apixaban (3.8%), cilostazol (2.5%), prasugrel (2.5%), dabigatran (2.5%), ethyl icosapentate (3.8%), sarpogrelate (1.2%), and ticlopidine (1.2%). Several patients were simultaneously administered multiple antithrombotic agents, reflecting the real‐world complexity of ATT management.

### 3.3 Intraoperative Findings and Surgical Outcomes

Before PSM, patients receiving ATT showed significantly longer operation times (317 [222–394] vs. 270 [195–377] minutes; *p* = 0.010), greater blood loss (400 [108–1061] vs. 300 [75–822] mL; *p* = 0.044), and a higher incidence of Clavien–Dindo ≥ grade III complications (31.5% vs. 19.9%; *p* = 0.008). The frequencies of hemorrhagic complications (5.5% vs. 1.8%; *p* = 0.022), thromboembolic events (6.3% vs. 2.0%; *p* = 0.006), and PHLF (17.3% vs. 8.0%; *p* = 0.003) were also significantly elevated in the ATT group. Postoperative mortality was observed in two patients in the ATT group (1.4%) and in one patient in the non‐ATT group (0.2%). These imbalances underscore the need for matching and adjustments in the subsequent analyses.

After PSM, the median operation time was significantly longer in the ATT group (318 [226–383] vs. 250 [188–369] min; *p* = 0.019). Although the median blood loss was modestly higher (355 vs. 300 mL), the difference was not statistically significant (*p* = 0.460). Transfusion requirements were similar between groups (ATT, 19.4% vs. non‐ATT, 23.5%; *p* = 0.602). No significant difference was noted in the incidence of severe postoperative complications (Clavien‐Dindo≥grade III): 32.7% in the ATT group vs. 22.4% in the non‐ATT group (*p* = 0.150). However, there was a trend toward a higher incidence of both bleeding and thrombotic complications in the ATT group. PHLF (ISGLS grade B or C) occurred in 16.3% of ATT patients versus 13.3% of non‐ATT patients (*p* = 0.688), and the median postoperative hospital stay was slightly longer in the ATT group (13 vs. 12 days; *p* = 0.050), suggesting delayed recovery in some patients (Table 2).

**TABLE 2:** Operative findings and postoperative complications before and after propensity score matching.

Variables	Non‐antithrombotic therapy (*n* = 609)	Antithrombotic therapy (*n* = 140)	*p*‐value	Non‐antithrombotic therapy (*n* = 98)	Antithrombotic therapy (*n* = 98)	*p*‐value
Operation time (min)	270 [195, 377]	317 [222, 394]	**0.010**	250 [188, 369]	318 [226, 383]	**0.019**
Bleeding (mL)	300 [75, 822]	400 [108, 1061]	**0.044**	300 [100, 830]	355 [135, 808]	0.460
Transfusion	109 (19.9)	31 (24.4)	0.350	23 (23.5)	19 (19.4)	0.602
CD ≥ III	109 (19.9)	40 (31.5)	**0.008**	22 (22.4)	32 (32.7)	0.150
Hemorrhagic complications	10 (1.8)	7 (5.5)	**0.022**	3 (3.1)	6 (6.1)	0.497
Thrombotic complications	11 (2.0)	8 (6.3)	**0.006**	4 (4.1)	8 (8.2)	0.372
PHLF (B or C)	44 (8.0)	22 (17.3)	**0.003**	13 (13.3)	16 (16.3)	0.688
Postoperative hospital stay	11 [8, 17]	13 [10, 23]	**0.019**	12 [9, 18]	13 [10, 23]	**0.050**

*Note:* Data are expressed as the median [IQR] and number (percent). Left columns: unmatched cohort; right columns: matched cohort (ATT vs. non‐ATT). Bold values denote statistical significance at the *p* < 0.05 level.

Abbreviations: CD, Clavien–Dindo classification; PHLF, Post hepatectomy liver failure.

### 3.4 Perioperative Complication Profiles by ATT Regimen: Hemorrhage, Thromboembolism, and Liver Failure

**FIGURE 1:**
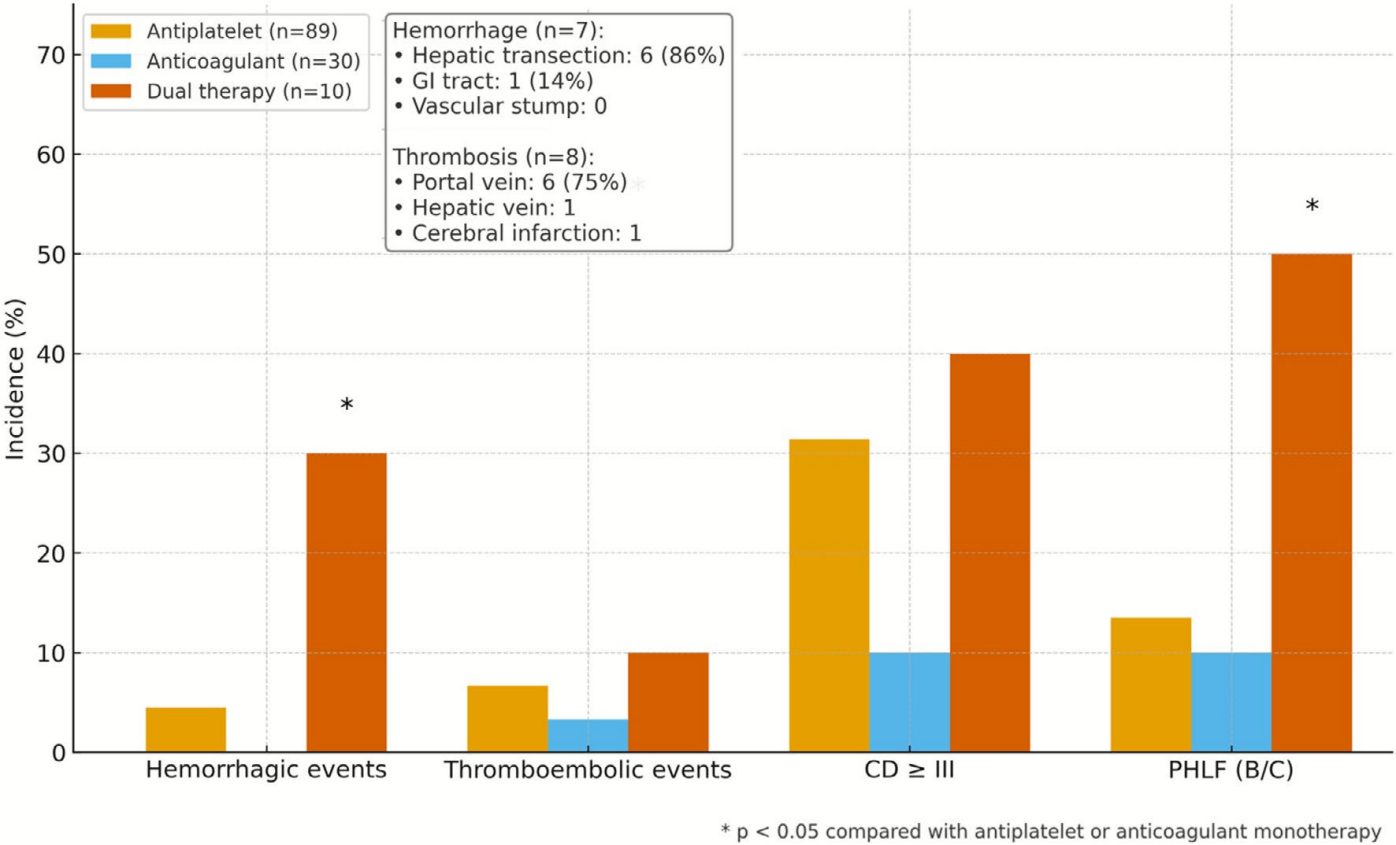
Incidence of Major Perioperative Complications According to Antithrombotic Regimen. Bar graph illustrating the incidence of major perioperative complications—hemorrhagic events, thromboembolic events, severe postoperative complications (Clavien–Dindo [CD] grade≥III), and post‐hepatectomy liver failure (PHLF; grade B or C)—according to the type of antithrombotic therapy administered. Patients receiving dual therapy (a combination of anticoagulant and antiplatelet agents) exhibited the highest complication rates across all categories. This figure underscores the elevated perioperative risk associated with dual antithrombotic therapy compared to monotherapy regimens.

In the ATT cohort (*n* = 140), global comparisons among the three regimens demonstrated significant differences in the incidence of hemorrhagic complications, Clavien–Dindo≥grade III complications, and PHLF, whereas no significant differences were observed in thromboembolic events, transfusion requirement, intraoperative blood loss, operative time, or length of postoperative hospital stay (Table 3). Subgroup analysis demonstrated distinct complication profiles based on regimen type. Hemorrhagic complications were most frequent among patients receiving dual therapy (antiplatelet plus anticoagulant), occurring in 30.0% (3 of 10), compared with 4.5% (4 of 89) in the antiplatelet group and 0% (0 of 30) in the anticoagulant group. These differences remained statistically significant in Bonferroni‐adjusted post hoc pairwise comparisons between dual therapy and either antiplatelet or anticoagulant monotherapy, indicating that patients on dual therapy are particularly susceptible to perioperative bleeding (Table 3, Figure 1).

**TABLE 3:** Subgroup analysis of perioperative outcomes according to antithrombotic regimen.

Variables	Antiplatelet monotherapy (*n* = 89)	Anticoagulant monotherapy (*n* = 30)	Dual therapy (*n* = 10)	Global *p*‐value*	Adjusted *p*‐value^†^ (Dual vs. Antiplatelet)	Adjusted *p*‐value^†^ (Dual vs. Anticoagulant)
Operation time (min)	335 [228, 395]	282 [204, 373]	226 [210, 256]	0.121	—	—
Bleeding (ml)	467 [171, 1092]	330 [37, 1184]	200 [117, 265]	0.223	—	—
Transfusion	25 (28.1)	4 (13.3)	3 (30.0)	0.242	—	—
CD ≥ III	28 (31.4)	3 (10.0)	4 (40.0)	**0.031**	1.000	0.086
Hemorrhagic complications	4 (4.5)	0 (0)	3 (30.0)	**0.009**	**0.022**	**0.018**
Thrombotic complications	6 (6.7)	1 (3.3)	1 (10.0)	0.580	—	—
PHLF (ISGLS grade B or C)	12 (13.5)	3 (10.0)	5 (50.0)	**0.017**	**0.002**	**0.024**
Postoperative hospital stay	13 [10, 22]	12 [9, 16]	19 [13, 33]	0.198	—	—

*Note:* Data are expressed as the median [IQR] or number (percent). Bold values denote statistical significance at the *p* < 0.05 level.

Abbreviations: CD, Clavien–Dindo classification; ISGLS, International Study Group of Liver Surgery; PHLF, Post hepatectomy liver failure.

* Global *p*‐values were calculated using the chi‐square test for categorical variables and the Kruskal–Wallis test for continuous variables.

^†^ Pairwise *p*‐values were calculated using Fisher's exact test for categorical variables and the Wilcoxon rank‐sum test for continuous variables, and were adjusted for two planned comparisons (dual therapy vs. antiplatelet monotherapy and dual therapy vs. anticoagulant monotherapy) using Bonferroni's method.

Among the seven hemorrhagic events observed in the ATT cohort, the hepatic transection plane was the most common bleeding site (6 of 7, 85.7%), followed by the gastrointestinal mucosa (1 case). Notably, no hemorrhage was attributed to vascular stump bleeding. Interventional radiology was required in three patients for hemostasis; none required surgical reintervention. Thromboembolic events were observed in 10.0% (1 of 10) of patients receiving dual therapy, 6.7% (6 of 89) in the antiplatelet group, and 3.3% (1 of 30) in the anticoagulant group; however, the global comparison did not reveal a statistically significant difference among the three regimens, and pairwise differences were likewise not significant (Table 3). Among the eight thromboembolic events, portal vein thrombosis accounted for the majority (6 of 8, 75%), while one case each of hepatic vein thrombosis and cerebral infarction was observed. All thromboembolic events were classified as Clavien–Dindo grade III or higher and required anticoagulation, thrombolysis, or intensive care. These findings suggest that complications in patients on ATT tend to localize to specific anatomical and vascular regions, with the portal and hepatic circulation being particularly vulnerable.

PHLF (grade B or C) was most frequent in the dual therapy group, occurring in 50.0% (5 of 10), compared to 13.5% (12 of 89) in the antiplatelet group and 10.0% (3 of 30) in the anticoagulant group. Consistent with the global test, Bonferroni‐adjusted pairwise analyses confirmed that dual therapy was associated with a significantly higher incidence of PHLF than either antiplatelet or anticoagulant monotherapy, whereas no significant difference was observed between the two monotherapy regimens (Table 3). Similarly, the rate of severe postoperative complications (Clavien–Dindo≥grade III) was highest in the dual therapy group at 40.0% (4 of 10). Although the global comparison was statistically significant for this outcome, the Bonferroni‐adjusted pairwise comparisons suggested only a trend toward higher rates of severe complications in patients receiving dual therapy. To further elucidate the determinants of PHLF, we conducted univariable and multivariable logistic regression analyses in the entire cohort. In the multivariable model, biliary reconstruction, anatomical resection, intraoperative transfusion, and dual antithrombotic therapy were independently associated with PHLF (ISGLS grade B/C), with dual therapy showing the strongest association (adjusted OR 21.40, 95% CI 4.89–93.40; Table 4). Although the wide confidence interval reflects the limited number of patients receiving dual therapy, these findings suggest that the markedly high incidence of PHLF in this subgroup cannot be explained solely by surgical complexity and support the need for meticulous perioperative management in patients on dual ATT.

**TABLE 4:** Uni‐ and multivariable logistic regression analysis for predictors of post‐hepatectomy liver failure (ISGLS grade B/C).

		Univariate			Multivariate	
	OR	95% CI	*p*‐value	OR	95% CI	*p*‐value
Sex						
Male	1.99	(1.02–3.88)	**0.042**	1.57	(0.76–3.21)	0.220
Albumin						
< 4.0 gdL	2.04	(1.21–3.45)	**0.007**	1.65	(0.91–2.96)	0.097
Surgical approach						
Open laparotomy	2.74	(1.56–4.79)	**< 0.001**	0.86	(0.41–1.80)	0.693
Biliary reconstruction						
Yes	5.38	(2.62–11.0)	**< 0.001**	2.97	(1.31–6.73)	**0.009**
Liver resection procedure						
Anatomical resection	4.64	(2.62–8.21)	**< 0.001**	2.71	(1.45–5.09)	**0.002**
Operation time (min)						
> 240 min	4.22	(2.12–8.41)	**< 0.001**	2.03	(0.88–4.66)	0.097
Bleeding (mL)						
> 500 mL	5.10	(2.91–8.95)	**< 0.001**	2.25	(0.99–5.10)	0.052
Transfusion						
Yes	4.72	(2.82–7.88)	**< 0.001**	2.02	(1.05–5.10)	**0.035**
Dual antithrombotic therapy						
Yes	10.70	(3.02–38.1)	**< 0.001**	21.40	(4.89–93.40)	**< 0.001**

*Note:* Bold values denote statistical significance at the *p* < 0.05 level.

Taken together, these findings indicate a disproportionate burden of hemorrhagic, thromboembolic, and hepatic dysfunction‐related complications in patients receiving dual ATT. Although limited by a small number of cases, the combination of global and post hoc analyses underscores the importance of regimen‐specific perioperative risk stratification and vigilant management in this high‐risk subgroup (Table 3).

## 4 Discussion

This propensity score‐matching study revealed two key findings. First, ATT, when appropriately managed with standardized perioperative protocols, did not significantly increase the risk of major intraoperative bleeding, transfusion, or overall postoperative morbidity in patients undergoing hepatectomy. Second, among patients receiving ATT, dual therapy, defined as the concurrent use of antiplatelet and anticoagulant agents, was associated with markedly higher rates of hemorrhagic events, thromboembolic complications, and PHLF, identifying these patients as a high‐risk subgroup.

These findings contribute to a more nuanced understanding of the perioperative risk in ATT recipients, suggesting that while liver resection is generally safe in patients receiving antithrombotic agents, dual therapy demands heightened caution and individualized management. Despite longstanding concerns regarding perioperative bleeding in ATT recipients, particularly during major abdominal surgery, our data suggest that hepatectomy can be safely performed using modern surgical techniques and careful coordination [8, 9, 10].

Historically, ATT has been routinely withheld to mitigate bleeding risk [11, 12]; however, interruption of therapy has been associated with increased thromboembolic events, including stroke, myocardial infarction, and venous thromboembolism [2, 13, 14, 15]. Several recent studies have specifically examined the safety of hepatectomy or hepatobiliary–pancreatic surgery in patients receiving antiplatelet or anticoagulant monotherapy [3, 16, 17, 18, 19]. In these reports, continuation of low‐dose aspirin or other antithrombotic agents during abdominal surgery, including liver resection, did not increase intraoperative blood loss, transfusion requirements, or postoperative hemorrhagic complications compared with patients not receiving antithrombotic therapy, suggesting that routine interruption of antiplatelet therapy may be unnecessary in carefully selected candidates for hepatectomy. Consistent with these findings, a recent systematic review [20] also concluded that liver resection in patients on chronic ATT, including continued aspirin monotherapy, can be performed safely without an increase in bleeding complications. Our findings are broadly consistent with these data for patients receiving antithrombotic monotherapy, but further extend the existing evidence by suggesting that, in our cohort, patients on dual ATT appeared to constitute a distinct high‐risk subgroup characterized by higher rates of hemorrhagic, thromboembolic, and PHLF‐related complications. To our knowledge, no prior report has specifically analyzed the perioperative risks associated with dual ATT and the concomitant use of both antiplatelet and anticoagulant agents. This is particularly important, as dual therapy is increasingly being encountered in clinical practice, especially among elderly patients with complex cardiovascular comorbidities.

In our cohort, patients on dual therapy experienced significantly higher rates of hemorrhage (30%), thromboembolism (10%), Clavien–Dindo ≥ grade III complications (40%), and PHLF (50%) compared with monotherapy. These complication rates substantially exceeded those of patients receiving antiplatelet or anticoagulant monotherapy and were statistically significant, indicating that dual‐therapy recipients represent a particularly vulnerable group. As shown in the multivariable logistic regression analysis (Table 4), biliary reconstruction, anatomical resection, intraoperative transfusion, and dual antithrombotic therapy were independently associated with PHLF (ISGLS grade B/C), with dual therapy showing the strongest association. Although the wide confidence interval for dual ATT inevitably reflects the small number of patients and the likelihood that these patients had a greater baseline burden of cardiovascular and thromboembolic disease, the markedly high incidence of PHLF in the dual‐therapy subgroup is unlikely to be explained solely by surgical complexity or baseline risk profile. These findings support the need for meticulous perioperative management and careful risk stratification in patients on dual ATT.

The operative time was significantly longer in patients on ATT, likely reflecting increased intraoperative vigilance. Blood loss and transfusion rates were comparable, indicating that standardized ATT protocols support safe surgical outcomes. This aligns with studies reporting that the continuation of low‐dose aspirin or DOACs does not increase the bleeding risk in gastrointestinal or urologic surgery [3, 16, 17]. Postoperative complications, although slightly more frequent in patients receiving ATT, were not statistically significant. Most hemorrhages originate from the hepatic transection plane and are managed effectively with interventional radiology, supporting the safety of ATT‐related bleeding [18]. Thromboembolic events, particularly portal vein thrombosis, although infrequent, were more common in the ATT group. Delayed ATT resumption or a lack of bridging therapy may have contributed [19, 21].

Polypharmacy with dual ATT (e.g., aspirin plus DOAC or warfarin) may contribute to the increased risk of adverse events, in combination with the high baseline cardiovascular and thromboembolic burden in these patients, in line with the known bleeding and thrombotic risks associated with such combinations [22]. Therefore, our findings support a more cautious perioperative strategy for patients on dual therapy, including careful preoperative risk stratification and potential modification of ATT regimens in selected cases.

Monotherapy with low‐dose aspirin appears safe, as supported by randomized trials and meta‐analyses, showing no significant bleeding risk or possible thromboembolic protection [17, 23, 24]. In addition, the slightly elevated incidence of PHLF in patients on ATT may reflect impaired hepatic perfusion following anticoagulation therapy. Maehira et al. identified alanine aminotransferase levels, prothrombin time, and remnant liver volume as predictors of PHLF, underscoring the need for individualized preoperative assessment [25, 26, 27]. Clinically, these findings highlight the importance of tailoring perioperative ATT management. Selective continuation or early resumption may be preferable for patients with high thromboembolic risk. Multidisciplinary coordination and adherence to the JCS [1], CHEST [6], and ESGE [3] are essential for risk optimization.

The limitations of this study include its retrospective, single‐center design and relatively small number of patients receiving dual therapy (*n* = 10). Although some trends in the dual‐therapy subgroup reached statistical significance, this analysis is statistically underpowered, and these findings should therefore be interpreted with caution. Validation in larger multicenter cohorts is required. Another important limitation is that specific indications for ATT, such as coronary artery disease, atrial fibrillation, or prior ischemic stroke, were not included as covariates in the propensity score model, and residual confounding by indication therefore cannot be excluded. In particular, patients receiving dual ATT may have had a greater burden of baseline cardiovascular and thromboembolic risk, and part of the observed increase in complications in this subgroup may thus reflect their underlying condition rather than the effect of dual therapy alone. Additionally, the lack of long‐term follow‐up of thromboembolic outcomes limits the evaluation of late complications.

In conclusion, hepatectomy can be safely performed in patients on ATT using standardized protocols. However, patients receiving dual ATT appeared to have higher rates of hemorrhage, thromboembolism, and PHLF, suggesting that this regimen should be regarded as potentially high risk. Our findings emphasize the need for increased perioperative care and individualized management in this subgroup. Prospective multicenter studies are needed to establish optimal strategies for patients requiring dual ATT.

## Author Contributions

**Haruki Mori:** conceptualization, methodology, writing – original draft, writing – review and editing, investigation, data curation, validation, formal analysis. **Hiromitsu Maehira:** resources, supervision, validation. **Nobuhito Nitta:** validation, resources. **Takeru Maekawa:** resources. **Toru Miyake:** validation. **Sachiko Kaida:** resources. **Masatsugu Kojima:** resources. **Katsushi Takebayashi:** resources. **Takeshi Sonoda:** resources. **Masaji Tani:** supervision, resources.

## Funding

The authors have nothing to report.

## Ethics Statement

This study was approved by the Human Ethics Review Committee of Shiga University of Medical Science (approval number: 2017–170) and conducted in accordance with the principles of the Declaration of Helsinki.

## Consent

Informed consent was obtained from all patients or their family members during the outpatient visits.

## Conflicts of Interest

The authors declare no conflicts of interest.

## Supporting information


**Data S1:** Perioperative management protocol for antithrombotic therapy.
