# Supplementary material for: Elevated Risk of Complications in Patients Receiving Dual Antithrombotic Therapy Undergoing Hepatectomy: A Single‐Center Audit of 749 Cases
Source: Ann Gastroenterol Surg. 2025 Dec 29;10(3):883–92. doi: 10.1002/ags3.70160 (PMC13178271; doi:10.1002/ags3.70160)
Supplement: Supplementary file 1 — Data S1: Perioperative management protocol for antithrombotic therapy. [file AGS3-10-883-s001.docx]

*Perioperative management protocol for antithrombotic therapy*

In patients with a high thromboembolic risk, low-dose aspirin is generally continued throughout the perioperative period. Other antithrombotic agents were withheld according to the standard cessation intervals based on drug-specific pharmacokinetics and individual risk profiles. Warfarin was typically discontinued 3–5 days prior to surgery, with perioperative heparin bridging employed selectively based on thrombotic risk. Direct oral anticoagulants (DOACs), including edoxaban, apixaban, and rivaroxaban, were discontinued 24–72 hours before surgery, depending on renal function and bleeding risk. Dabigatran was stopped 2–4 days preoperatively based on the estimated creatinine clearance. Regarding antiplatelet agents, clopidogrel, prasugrel, and ticlopidine were discontinued 5–7 days before surgery. Cilostazol, sarpogrelate, and ethyl icosapentate were usually discontinued 2–5 days before surgery at the discretion of the attending physicians, considering the bleeding risk and urgency of the procedure. In the postoperative period, antithrombotic agents were generally resumed on postoperative day 1 or 2 in patients without evidence of active bleeding or significant hemorrhagic tendency. The decision regarding the timing and selection of agents for resumption was made through multidisciplinary consultation, primarily involving the surgical and cardiology teams, considering the patient’s thromboembolic risk, bleeding profile, and overall clinical course.
